# Pure mucinous breast carcinoma in a 25-year-old female, a case report

**DOI:** 10.1016/j.ijscr.2018.10.009

**Published:** 2018-10-17

**Authors:** Irean Garcia-Hernandez, Carlos A. Lopez-Garcia, Servando Cardona-Huerta, Rocio Ortiz-Lopez, Nydia Paulina Herrera-Rios, B. Kanagusico-Elguezabal, Esteban-Zubero Eduardo, Gabriela Sofia Gomez-Macias

**Affiliations:** a Tecnologico de Monterrey, Hospital San Jose, Servicio de Patología, Mexico; b Tecnologico de Monterrey, Hospital San Jose, Centro de Tratamiento de Mama, Mexico; c Tecnologico de Monterrey, Escuela de Medicina y Ciencias de la Salud, Mexico; d Tecnologico de Monterrey, Hospital San Jose, Centro diagnóstico de Imagen diagnostica de mama, Mexico; e Rioja Salud, Hospital San Pedro, Departamento de Urgencias, Logroño Spain; f UANL, Facultad de Medicina, Hospital Universitario, Mexico

**Keywords:** Breast cancer, Mucinous carcinoma, Young patient

## Abstract

**Introduction:**

Mucinous carcinoma is a variant of invasive breast carcinomas representing 2% of them. These tumors frequently develop in postmenopausal females; it is a rare histological variant in young patients.

**Case presentation:**

A 25-year-old female refers a slow growth mass of 2 years of evolution. Excisional biopsy reveals a pure mucinous carcinoma with positive hormone receptors and negative HER2. She was treated with hormone therapy and surgical resection.

**Discussion:**

Mucinous carcinoma is a rare variant reported in young patients. Many series report that is frequently found in postmenopausal patients. We present a case of a pure mucinous carcinoma in a 25-year-old female with the importance of being a low-frequency malignancy in young patients.

**Conclusion:**

Due to its benign course, it is important to know that this lesion can also present in young patients. The importance underlies in the multidisciplinary management at the right time in a proper way.

## Highlights

• Young patient.
• Mucinous carcinoma.
• Prognosis.
• Hormone receptor.

## 1 Introduction

Mucinous breast carcinoma is a rare entity, represents 2% of the invasive breast carcinomas [1]. To classify it as pure the mucinous compound must be up to 90% of the tumor. It is usually seen in postmenopausal patients from 31 to 88-years-old (with an average of 67) [2]. Fewer cases have been reported in younger patients with an early onset of 31-years-old [3]. This carcinoma represents an excellent prognostic histological variant [1] to its positivity to ER and PR, and negativity to HER receptors [4]. Since it is a rare histological variant in young patients, we report a 25-year-old Mexican patient with a pure mucinous breast carcinoma. This case report has been described under the SCARE criteria [16]

## 2 Case report

A 25-year-old Mexican female with family history of ovarian cancer at her maternal side and personal history of a mass on her right breast clinically and radiologically diagnosed as fibroadenoma 2 years ago. The same mass had a growth of 2 cm so an incisional biopsy was performed at another institution. This was positive for mucinous carcinoma, and the patient was referred to our institution for treatment and follow up.

**Fig. 1:**
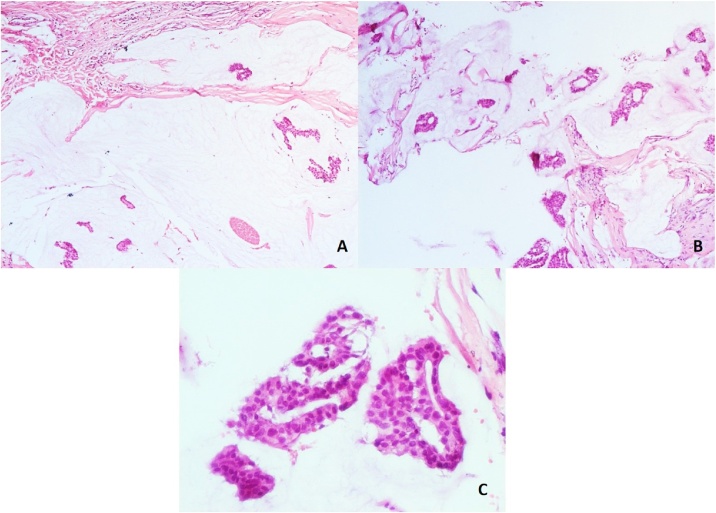
Incisional biopsy (A, B) Panoramic view of the tumor is composed of multiple groups of neoplastic cells immerse on extracellular mucin (hematoxylin-eosin 4X). (C) Mucin lakes with cellular characteristics of the tumor (40X).

At our institution in the biopsy slides revision, we found a tumor composed of mucinous lakes with numerous tumor cells of medium to large size with a moderate amount of eosinophilic cytoplasm confirming the presence of pure mucinous carcinoma. We could not identify the tumor size and surgical margins by no prior radiological and pathological report. [Fig. 1]. The tumor was classified as a Low grade tumor (G1) based on the Scarff Bloom Richardson scale. We did not observe perineural or vascular invasion. On immunohistochemistry the results were 100% and 90% positive for estrogen and progesterone receptors respectively with high intensity, and HER2 receptors negative.

**Fig. 2:**
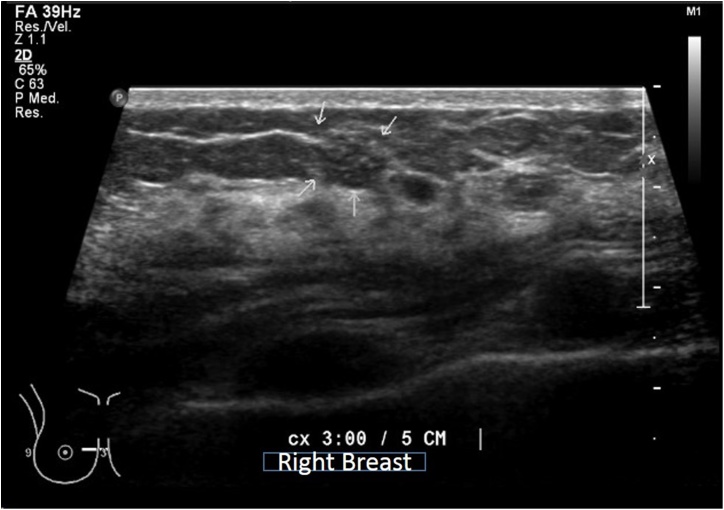
Avascular distortion, and adjacent to this site were found 2 homogeneous masses and a heterogeneous mass with microcysts (white arrows).

On physical examination a surgical injury was seen. Upon palpation, this zone felt indurated, edematous and presented as a rough irregular texture. No other lesions were found. An ultrasound was performed, showing avascular distortion and a heterogeneous mass with microcysts (white arrows) [Fig. 2]. MRI showed a hypointense irregular mass that indicates a mild enhancement in contrast sequences and a hyperintense sequence in SPAIR. The evaluation of the lymph nodes was not suspicious for metastasis.

With the radiological suspicious of residual tumor and pathological findings accomplished at our institution a partial mastectomy and a sentinel lymph node biopsy was performed. Pathological findings showed a residual mass of 25 mm of pure mucinous carcinoma with negative surgical margins. Sentinel lymph node was negative for metastasis.

Molecular screening with EndoPredict clinic showed a score of 2.7 classifying it as a low risk, reason why this patient was treated with tamoxifen because her positivity to receptors and low risk showed in molecular assay. The clinical and pathological stage IB was confirmed.

## 3 Discussion

Mucinous carcinomas represent 2% of invasive breast carcinomas, characterized by the production of the extracellular mucin within the tumoral cells. The tumor should have 90% of this characteristics to be considered as a pure mucinous carcinoma.

These tumors frequently developed in postmenopausal patients with an average of 67-years-old [2,5]. Di Salverno et al. evaluated the incidence in 11,422 patients with pure mucinous carcinoma having an average of 68.3-years-old with a range of 25 to 85-years-old. Most of the patients had 65-years-old or older (66.3%) and 18.2% younger than 55-years-old having the youngest patient 25-years-old [7,8]. The incidence in younger patients represents less than 1% having the youngest patient less than 35-years-old [5]. Lowman M et al. review consists of a series of 3482 patients of pure mucinous carcinomas (2.2% of all tumors reviewed) of these reported patients older than 70-years-old corresponding to the 59%, from 50 to 69 years-old corresponding to the 28% and younger than 50-years-old corresponding to the 13% [6]. Komenaka et al. realized a review of 65 cases with an age range from 31 to 65 years old, with an average age of 67-years-old from this 85% (55 to 65-years-old) were postmenopausal patients [9].

Most of the palpable lumps may be a challenge for radiologists. Mucinous carcinomas could be misdiagnosed as benign lesions because of slow growth phase and macroscopically well circumscribed margins [3,9]. These tumors are classified as low-grade tumors. Immunohistochemistry showed positivity for estrogen and progesterone receptors (81%) and negativity for Her2 (93%), there has been reported less than 5% of patients with lymph node metastases [4,11,13]. This means a favorable prognosis for the disease [13,14].

We present a case of a pure mucinous carcinoma in a 25-year-old Mexican female with the importance of being a low-frequency malignancy reported in patients younger than 30-years-old in the literature. It is important the correct and differential diagnosis in breast masses at young patients because of the frequency of benign lesions (56%) [10,12,13], and because mucinous carcinoma as malignancy is an oddity histological variant not commonly seen in young patients [15].

## 4 Conclusion

Pure mucinous carcinoma represents a histological variant commonly reported in postmenopausal patients. To our knowledge, there are a very few reported cases of this histological variant in young patients (<1%) [9] being this patient reported with the lowest age in the literature. The importance of this case report underlies in the multidisciplinary management of this malignancy at the right time in a proper way.

## Conflicts of interest

We don´t have any conflicts of interest

## Sources of funding

We don´t have any sources in my research

## Ethical approval

Our study is exempt from ethnical approval by our institution.

## Consent

Written informed consent was obtained from the patient for publication of this case report and accompanying images. A copy of the written consent is available for review by the Editor-in-Chief of this journal on request

## Author contribution

Garcia Hernandez Irean: Study concepts, study design, manuscript editing and review.

Lopez Garcia Carlos A.: Study concepts, study design, manuscript editing and review.

Cardona Huerta Servando: Data acquisition, manuscript editing.

Ortiz Lopez Rocio: Data acquisition, manuscript editing.

Herrera Rios Nydia P.: Data acquisition manuscript preparation

Kanagusico Elguezabal B.: Data acquisition, manuscript preparation.

Eduardo Esteban Zubero: Manuscript review, manuscript editing.

Gomez Macias Gabriela Sofia: Data acquisition, manuscript editing manuscript review.

## Registration of research studies

This study does not require declaration.

## Guarantor

Gabriela Sofia Gomez Macias

## Provenance and peer review

Not commissioned, externally peer reviewed.
